# A Home Disaster Preparedness Intervention Study with Korean American Residents in New York City

**DOI:** 10.1007/s11524-025-00974-z

**Published:** 2025-03-31

**Authors:** Tara Heagele, JungMoon Hyun, So-Hyun Park, William Ellery Samuels, Jin Young Seo

**Affiliations:** 1 Hunter-Bellevue School of Nursing, Hunter College, the City University of New York, New York, NY USA; 2 Speech, Language, & Hearing Sciences, Auburn University, Auburn, USA; 3 Ewha Womans University, Seoul, South Korea

**Keywords:** Community, Disaster planning, Disasters, Elderly, Emergency preparedness, Emigrants and immigrants, Preparedness, Hazard, Health surveys, Intervention study, Kit, Medically frail, Public health, Readiness, Vulnerable populations

## Abstract

This study investigated the effectiveness of the Nurses Taking on Readiness Measures (N-TORM) intervention in improving disaster preparedness of Asian immigrant residents in New York City. N-TORM is an innovative, nurse-led, household disaster preparedness educational intervention provided to community members to help them create their disaster-related evacuation and communication plans, build disaster supply kits, and understand community resources available to assist residents before, during, and after disasters. The aims of this controlled before-after intervention study were to (a) pilot test the Korean translation of the Household Emergency Preparedness Instrument (K-HEPI), (b) evaluate the effectiveness of N-TORM to increase household disaster preparedness knowledge and behavior, and (c) describe the factors most necessary to maintain and expand N-TORM. Participants in the experimental group (*N* = 200) demonstrated significantly greater pre-to-post improvement in disaster preparedness compared to those in the control group (*N* = 199), as measured by the K-HEPI (effect on total General Preparedness scores, *β* = 1.66, *SE* = 0.105, *p* < .001). The Korean community organization that was involved in this study continues to offer N-TORM to its members, demonstrating the intervention’s sustainability. Further testing of the K-HEPI and the intervention in longitudinal studies is recommended to explore potential associations between disaster preparedness and outcomes such as survival without rescue, prevention of injury, or acute exacerbations of chronic illnesses. Replicating the intervention across different immigrant populations is essential, and data from these studies can inform policy development to better address the needs of immigrant communities before, during, and after disasters. ClinicalTrials.gov Identifier: NCT0554478. Hunter College of City University of New York Protocol Record 2022–0542-Hunter, Korean Translation and Validation of the K-HEPI by a Phase 1 Feasibility Study in NYC, is registered and posted on the ClinicalTrials.gov website.

**Supplementary Information:**

The online version contains supplementary material available at 10.1007/s11524-025-00974-z.

## Introduction

Residents of New York City (NYC) are at risk for adverse events to their homes and communities from extreme weather and disasters such as coastal storms, flooding, droughts, earthquakes, extreme heat, winter storms, poor air quality, and non-natural hazards [1]. The reality of these risks came to fruition on the stormy night of September 1, 2021, when the effects of Hurricane Ida arrived in NYC. This coastal storm caused record rainfall and widespread flooding. Thirteen people died in NYC that night, 11 of whom drowned when their basement-level homes flooded. The majority of these deaths transpired in low-income Asian immigrant communities [2]. Immigrant populations face additional, unique risks during extreme weather events compared to the rest of the population residing in the same geographical area due to such factors as limited English proficiency, minimal social connections beyond the immigrant community, and undocumented immigration status [3].

Following the tragedies experienced during Hurricane Ida, members of the Asian immigrant community in NYC stressed the need for household disaster preparedness education and community-focused interventions, which they requested from the study team. Before designing and implementing a disaster preparedness intervention, we conducted a review of the literature to determine what was already known about the household emergency preparedness and disaster experience of Asian immigrants in NYC, specifically between 2001 (post 9/11) and August 2022. The literature search strategies are found in the online supplemental material (Appendix).

There has been limited research on the disaster experiences of Asian immigrants compared to non-Asian immigrants in NYC. Among 15 articles identified in the search, two articles detailed the lack of English proficiency as a barrier to disaster relief assistance [4, 5]. Three articles detailed the effects of Hurricane Ida to Asian immigrants [6–8]. The data demonstrated increased mortality rate, property destruction of basement apartments, and need for disaster assistance from the Federal Emergency Management Agency, which the researchers attributed to language barriers, skepticism in the Asian immigrant community, and rising Asian hate crimes due to the coronavirus pandemic.

Both Chau et al. [4] and Zhang [9] analyzed data from the Social Vulnerability Index to assess older Asian immigrant populations and evaluate current disaster preparedness and planning across comparison populations in NYC. Chau et al. addressed limited English proficiency as a barrier to effective disaster communication as part of an indicator for avoidable hospitalizations [4]. Articles about extreme heat and the Asian immigrant populations demonstrated negative associations with mortality rate [10, 11].

There has also been little study of how and how well Asian immigrants in NYC have been educated about evacuation planning, communication strategies, and supply kits for disaster preparedness. Of the 37 articles identified, none specifically addressed disaster preparedness education for Asian immigrants in NYC. However, 10 articles were relevant to the general topic. Among these, five articles discussed disasters in NYC but made no mention of Asian immigrants [4, 12–15]. Six articles addressed psychological resilience or the psychological aftermath of various disasters but did not explore methods to mitigate these effects through disaster preparedness education [12–14, 16–18]. One article highlighted the lack of disaster preparedness education in Latino immigrant communities, further emphasizing the gap in addressing immigrant populations’ disaster-related risks [19].

Due to the lack of articles on our target population, we concluded that the Asian immigrant communities in NYC likely face challenges similar to those of other immigrant populations. Despite the significant size and diversity of Asian immigrant communities in NYC, there is a paucity of research on their disaster experiences, and, to the best of our knowledge, no studies have examined disaster preparedness interventions tailored for this population. This study fills a significant knowledge gap.

Following an instrument translation study [20] that supported the reliable and valid use of the Korean version of the Household Emergency Preparedness Instrument (K-HEPI) to measure and detect changes in level of household disaster preparedness of at-risk populations, we employed it in a controlled before-after study of the Nurses Taking on Readiness Measures (N-TORM) intervention conducted with Korean-speaking residents in NYC.

RQ 1: What is the baseline level of household disaster preparedness of the participants?

RQ 2: Is there a significant improvement in household disaster preparedness after participating in the intervention?

H 2.1: Members of households who participate in the N-TORM educational intervention will show significantly greater increases in K-HEPI scores than members of households that do not participate in the intervention.

RQ 3: What are the facilitators and barriers of community health workers (trained by nurses) delivering and participants receiving the N-TORM intervention?

H 3.1: Factors commonly identified as predictors of household disaster preparedness (e.g., history of chronic disease, age, and household structure) will be significantly associated with posttest levels of household disaster preparedness in both the experimental group (those who participated in the intervention) and the control group (those who did not participate).

H 3.2: Factors commonly found to be predictors of household disaster preparedness will significantly interact with whether or not participants complete the intervention, such that barriers will affect those who do not complete the intervention more than they affect those who do complete it.

## Methods

This study was approved as “exempt with limited review status” by Hunter College of the City University of New York (IRB protocol #2022–0542-Hunter) on September 9, 2022.

Paper consent forms were provided to in-person control-group participants and all experimental-group participants prior to data collection. For online control-group participants, an online consent form was provided via Qualtrics Survey software prior to data collection. There was no more than minimal risk for participation in this study.

Experimental-group participants received $30 compensation ($10 for the first data collection session and $20 for the follow-up) while control-group participants received $20 ($10 for each session). Participants were encouraged to use this compensation to purchase non-perishable food items that meet the household’s dietary needs for their disaster kit.

Potential participants were recruited from Korean Community Services of Metropolitan New York, Inc. (KCS), one of the largest community-based, social service organizations in the USA. KCS is dedicated to addressing the needs of the Korean American communities, helping them overcome economic, health, and social barriers to become independent and thriving members of the community [21]. KCS has seven locations across NYC’s Queens, Brooklyn, and Manhattan boroughs, offering a wide range of services that extend beyond community and immigration assistance.

Participants were recruited at the KCS community centers via both on- and off-line flyer distribution, including outreach efforts, such as health fairs, engagement with ethnic media (i.e., local Korean newspapers and radio stations), workshops at local libraries, partner organization facilities, and at Korean churches. Screening was conducted in-person, via telephone, or online, depending on how the potential participant contacted the study team.

One participant per household was eligible to participate. Inclusion criteria included being aged 18 years or older, fluent in Korean, and living independently within the New York metropolitan area. Participants with cognitive impairments were excluded due to their limited role in household disaster preparedness. Non-Korean-speaking individuals were excluded because this study was also a pilot test of the K-HEPI. Study team members screened participants for eligibility, delivered the recruitment script, obtained informed consent, and administered the demographic data form.

Recruitment and data collection for the study began in February 2024 and concluded in July 2024. Three KCS sites, which could accommodate large class sizes, were selected to host the experimental groups. To limit cross-contamination, recruitment for the control group was excluded from sites nearest to the intervention locations. Other potential data collection sites were contacted to determine their interest in participating. Participants were assigned to study groups based on the site through which they were recruited, and sites were blinded to which group their members were assigned to.

After the K-HEPI was translated and field tested [20], we utilized it in a controlled, single-blinded, non-randomized, before-after study to measure the level of household disaster preparedness of the target population before and after participating in the N-TORM educational intervention. We compared the K-HEPI scores of the experimental group and the control group at baseline and 1-month follow-up.

This was a phase I feasibility study for which we did not have prior knowledge of effect sizes, correlations of scores between time points, or estimations of any sphericities between time points. We therefore estimated that we would have a “small” effect (*f* = 0.1) [22] for differences between the two groups measured at two time points, that the inter-time correlation of K-HEPI scores would be moderately high (*r* = 0.5) and that there would not be significant sphericity. We assumed power (1 – *β*) is 0.8 and used *α* = 0.05 as the threshold for statistical significance. Given these parameters, we estimated needing a total of 350 participants for a repeated measures ANCOVA. To account for a potential attrition of 10%, we estimated needing 388 participants.

The primary outcome was the K-HEPI General Preparedness score, which was derived from a translation [20] of the 51-item English HEPI [23–25] that measures disaster preparedness actions, knowledge, and supplies of households. The K-HEPI questions are objective and assess what the respondent presently does or has for disaster preparedness in a dichotomous format. Respondents are asked to check which preparedness actions they have completed and what disaster supply kit items they have in their home; raw scores on the K-HEPI’s General Preparedness scale can range from 0 to 41. The General Preparedness scale is designed to be divisible into two subscales, Preparedness Actions and Planning and Disaster Supplies and Resources, that are themselves applicable to all households. The K-HEPI has three additional subscales (Special Actions Part 1, Special Actions Part 2, and Access and Functional Needs) that are only relevant to households with infants, children, pets, utility connections within the home, or with members who are dependent on prescription medications or eyeglasses, have a disability, are 65 years of age or older, or are pregnant.

Secondary outcomes included participants’ experience with the N-TORM intervention and the community health worker’s experience with implementation. Covariates included military status, history of chronic disease, disability status, predominant language proficiency, age, household composition or familial structure, ethnic/national origin identity, place, race, gender, education, employment, income, disaster risk perception, disaster preparedness self-efficacy, and prior disaster experience.

### Control Group

While the control group did not receive a disaster preparedness intervention, they may have been exposed to mass media campaigns in NYC. *After* their second data collection, participants received the Korean version of the two-page pocket guide, *Prepared New York: Disaster and Emergency Preparation* [26]. In-person participants received paper copies, while online participants were provided with a PDF link.

### Experimental Group

Nurses on the study team trained KCS community health workers to deliver the N-TORM intervention. One individual was selected as the instructor to ensure consistency in delivering the educational component. The instructor demonstrated competency by teaching back to the study team. She used a standardized presentation and handouts for all classes. To monitor intervention fidelity, study team members observed most N-TORM sessions and documented the instructor’s activities, including meeting frequency, content, and duration.

The experimental group received two, 1-h, in-person classes 2 to 4 weeks apart to help them develop their family evacuation and communication plans, identify community resources, and assemble a disaster supply kit. The instructor used a lecture and the Korean version of a booklet called *Ready New York: My Emergency Plan* [26] along with a disaster supply kit shopping list as handouts to educate the participants about the essentials of household disaster preparedness.

The first class began with instructing the participants to fill out the booklet during class and complete the remaining sections as homework. The class content complemented the booklet and the K-HEPI. The instructor reviewed different sections of the booklet and instructed the participants on how to write down their emergency plans. Participants wrote their medical history, medications, physicians, next of kin, pharmacy contact information, evacuation plan information, and emergency communication plan. The booklet included household disaster preparedness recommendations, evacuation zone information, a disaster supply kit contents list, local utility contact information, insurance coverage recommendations, shelter locations, and special needs and priority utility restoration registry information. Participants were instructed to keep their completed booklets as a reference for their household disaster preparedness plans and community disaster resources information.

During the second class, the instructor reviewed the participants’ booklets for completion (approximately 2 min per participant), addressed their questions on their evacuation and communication plans and disaster kits, and facilitated a 30-min group discussion to share disaster preparedness experiences and foster social support. The discussion was guided by participants’ progress with their booklet completion. For example, if a participant had not listed two emergency contacts, the instructor asked, “Is anyone willing to serve as the second emergency contact for a classmate? If so, please exchange contact information.” Similarly, if a participant had not recorded an emergency meeting place, she prompted, “What locations have others chosen as a meeting place in case of an emergency evacuation?” Finally, the instructor led a discussion on tips and techniques for creating the disaster supply kit by asking, “Has anyone created or started their disaster supply kit? What is your plan to complete it?”.

### Data Analysis

Descriptive statistics were reviewed prior to regression analyses examining the associations between the study variables. We tested differences in K-HEPI scores between the groups (control vs. experimental) and times (pre- vs. posttest) with hierarchical linear regressions with time nested within participants. Adding additional terms, like demographics, did not lead to stable final models and so were not included in these tests.

The reliability of the K-HEPI was measured by looking at the internal consistency of the items (ordinal *α* coefficients and item-total correlations). Confirmatory factor analysis was used to examine the factor structure of the K-HEPI and to compare it with that of the English version. Details on the psychometric data analysis are provided in Samuels et al. [20].

## Results

### Quantitative Data

The study achieved the desired sample size with 399 participants (199 in the control group and 200 in the experimental group). Among the control group, 6 participants (3%) did not complete the follow-up survey. In the experimental group, 17 participants (8.5%) did not attend the follow-up sessions.

Table 1 presents demographic and related results for members of the control and experimental groups. These tables indicate that there were several significant differences between participants assigned to the two groups. The differences in the counts in relationship types (*χ*^2^ = 29, *p* < 0.001), employment (*χ*^2^ = 81, *p* < 0.001), education (*χ*^2^ = 26, *p* < 0.001), and income (*χ*^2^ = 28, *p* < 0.001) were significantly different. Analyses of differences in K-HEPI scores between the control and experimental groups that included these additional terms did not converge, however, so they were not included in those tests.

**Table 1:** Characteristics of the control and experimental arm participants

Variable	Control, mean (*N*, *SD*)	Experimental, mean (*N*, *SD*)	*t*	*p*
Age	51.64 (199, 18.14)	66.00 (200, 12.95)	− 13.06	< .001
Years in current home	8.66 (199, 8.13)	13.10 (200, 11.73)	− 7.45	< .001
Years in current community	14.87 (199, 9.61)	21.64 (200, 13.86)	− 6.23	< .001
Years in the USA	23.25 (370, 11.36)	29.81 (199, 13.05)	− 8.03	.002
Variable	Control	Experimental	*X*^2^	*p*
Military	34 (17.1%)	27 (13.5%)	0.73	.392
Speaks English	37 (18.7%)	20 (10.0%)	5.43	.020
Speaks Korean at home	192 (96.5%)	195 (97.5%)	0.09	.763
Children in home	72 (36.2%)	36 (18.0%)	15.79	< .001
Adults in home	153 (76.9%)	80 (40.0%)	54.35	< .001
Older adults in home	88 (44.2%)	155 (77.5%)	45.01	< .001
English proficiency	77 (38.7%)	60 (30.0%)	28.51	< .001
Born in the USA	14 (7.0%)	2 (1.0%)	7.94	.005
Variable	Control	Experimental	*X*^2^	*p*
Takes medications	106 (53.3%)	144 (72.0%)	14.17	< .001
Needs medical equipment	19 (9.5%)	15 (7.5%)	0.31	.580
Has a disability	18 (9.0%)	25 (12.5%)	0.90	.342
Healthcare provider discussed disaster preparedness	29 (14.6%)	35 (17.5%)	0.44	.509
Home damage due to a disaster	16 (8.0%)	35 (17.5%)	7.18	.007
Disaster-related injury or illness	22 (11.1%)	30 (15.0%)	1.04	.307
Risk perception	28 (19.6%)	42 (21.0%)	4.29	.117
Self-efficacy	90 (45.2%)	85 (42.5%)	3.20	.020
Variable	Control (*N* = 199)	Experimental (*N* = 200)	*X*^2^	*p*
Home ownership			10.01	.007
• Own	55 (27.6%)	84 (42.0%)		
• Rent	138 (69.3%)	109 (54.5%)		
• Other	5 (2.5%)	7 (3.5%)		
• Not applicable	1 (0.5%)	0 (0.0%)		
Home structure			3.91	.419
• Detached single story	50 (25.1%)	56 (28.0%)		
• Multi, 1 or 2 stories	78 (39.2%)	63 (31.5%)		
• Multi, 3 or more stories	66 (33.2%)	75 (37.5%)		
• Mobile or manufactured home	1 (0.5%)	0 (0.0%)		
• Other	4 (2.0%)	6 (3.0%)		
• Not applicable	0 (0.0%)	0 (0.0%)		

Mean, number, and standard deviation and tests of differences in ages and years in home, community, and the USA for control and experimental-group participants. *X*^2^ tests compare counts between the control and experimental groups

Number and percent of control- and experimental-group participants responding “yes” to military experience, language fluencies, family members, household composition, and whether born in the USA

Number and percent of control- and experimental-group participants responding “yes” to health- and disaster-related items. *X*^2^ tests compare counts between the control and experimental groups

Number and percent of control- and experimental-group participants with various home ownership status and type of home. *X*^2^ tests compare counts between the control and experimental groups

#### Tests of Group Differences on K-HEPI Pre- and Posttest Scores

**Fig. 1:**
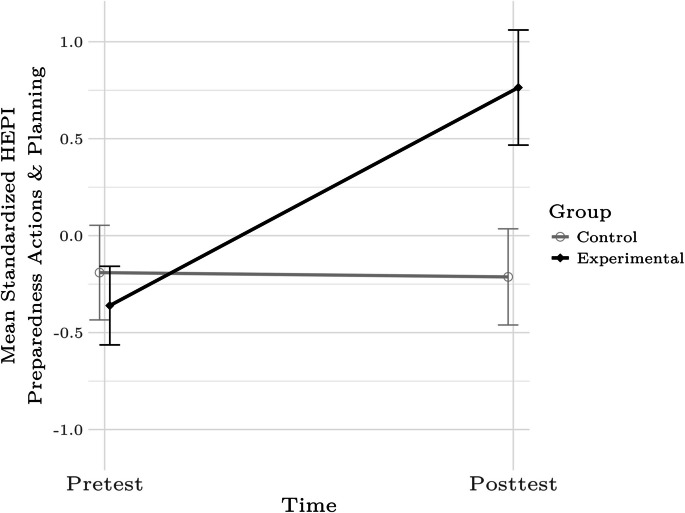
Mean standardized HEPI Preparedness Actions and Planning Subscale scores for the control and experimental groups at pre- and posttest. Error bars denote 95% confidence intervals. Figure was created using The R Project for Statistical Computing software

**Fig. 2:**
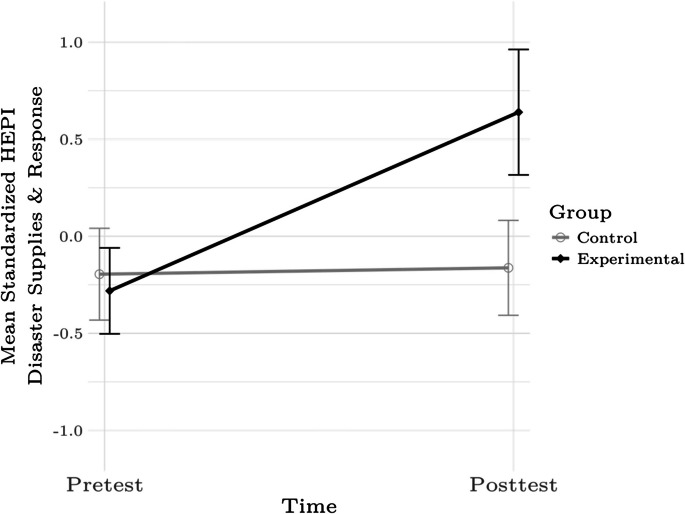
Mean standardized HEPI Disaster Supplies and Resources Subscale scores for the control and experimental groups at pre- and posttest. Error bars denote 95% confidence intervals. Figure was created using The R Project for Statistical Computing software

Figures 1 and 2 present the mean pre- and posttest K-HEPI General Preparedness subscores for the control and experimental groups. These figures suggest that there were significant differences for the two K-HEPI subscales that make up the General Preparedness score that is applicable to all households. Table 2 summarizes tests of these differences. Those who participated in the intervention realized greater pre-post gains in disaster preparedness than those who did not.

**Table 2:** Tests of differences in K-HEPI total and subscores between the experimental and control groups at pre- and posttest

HEPI subscore		*β*	*SE*	*p*
Preparedness Actions and Planning Subscale		1.29	0.103	< .001
Disaster Supplies and Resources Subscale		1.07	0.110	< .001
Special Actions Subscales	Total	0.25	0.114	.028
	Part 1	0.343	0.115	.003
	Part 2	− 0.05	0.111	.648
Access and Functional Needs Subscale		0.66	0.108	< .001

Significant terms indicate that the changes from pre- to posttest were reliably different between the groups; positive values indicate that the experimental-group participants realized greater pre-post differences than control-group participants

### Qualitative Data

Questions and comments from experimental-group participants during class sessions were documented for qualitative content analysis to assess whether modifications to the N-TORM intervention were needed. The most common question, asked four times, concerned where to purchase disaster kit supplies, particularly a hand-crank, battery-operated, solar-powered weather alert radio, with many participants reporting they do not shop online. Ultimately, participants were advised to ask family or friends to purchase the radio online for them. For future replication of the intervention with older adults, we recommend providing the radio as compensation.

In addition, an open-ended question on the participant experience survey elicited enthusiastic feedback supporting the intervention, along with some recommendations for improvement (Table 3, available online).

The community health worker who delivered the N-TORM intervention completed an implementation survey to evaluate her experience. The survey was delivered twice: initially after training but before conducting any participant education and again after all intervention sessions were completed. Pre-intervention feedback noted the “well prepared instructional materials,” the “teamwork of the cooperative staff,” and that it was “easier to distinguish the more important and less important points [of the presentation] because the researcher sent the English education video and [presentation] explanation in advance.” In her post-intervention survey, the primary challenges reported were participants unexpectedly changing their schedules and the difficulty of engaging “participants [who were] too old” to fully grasp the material. For cases involving age-related cognitive decline, we recommend inviting caretakers to participate in the intervention alongside these community members.

The community health worker recommended offering the N-TORM intervention regularly, stating that it “helped participants understand how to prepare for times of disaster” and that “after listening to the class, more participants became interested in disaster preparedness.” She also emphasized the value of the group discussion, noting that it enhanced participants’ desire to learn as a group.

## Discussion and Conclusion

Disasters, extreme weather events, public health emergencies, and humanitarian crises are becoming increasingly frequent and severe, disproportionately affecting immigrants residing in urban communities [27]. Many disaster-related injuries, illnesses, and deaths could be prevented through proactive measures such as evacuation route planning, family communication strategies, and stockpiling essential supplies (e.g., water, food, and medications) to sustain families during disruptions like power outages or shelter-in-place orders [23]. Healthcare providers, public health officials, emergency managers, community and faith-based organizations, and policymakers all play a critical role in equipping communities with knowledge and tools to mitigate disaster risks. This study contributes to the disaster preparedness literature by introducing a scalable educational intervention designed specifically for immigrant populations. Unlike interventions limited to a specific disaster type or geographical region, this model can be adapted to different communities based on their unique needs. By addressing gaps in preparedness among vulnerable urban populations, this study highlights a practical, community-based approach to enhancing disaster resilience in neighborhoods.

Participants in the experimental group realized greater pre-post gains in disaster preparedness than those in the control group, demonstrating the effectiveness of the N-TORM intervention. Furthermore, the KCS organization plans to continue offering the N-TORM intervention to all members beyond the study’s conclusion, supporting the sustainability and diffusion of the program.

Similar findings were reported in recent studies targeting disaster preparedness in immigrant populations. For example, a quasi-experimental study involving 165 Nepalese immigrants in Japan assessed the efficacy of an earthquake preparedness educational intervention [28]. The intervention included a 45-min lecture and discussion, a 10-min demonstration, and a 5-min question-and-answer session in the Nepali language. Results indicated significant increases in earthquake preparedness knowledge and practice immediately post-intervention and at 2- and 12-week follow-up. The study highlighted the importance of delivering interventions in immigrants’ native languages.

Another study examined the efficacy of disaster preparedness workshops in Alberta, Canada, with an immigrant community [29]. Workshop coordinators aimed to enhance disaster readiness through a 6-h workshop that included interactive presentations, hands-on learning, and community engagement activities. Results showed that the workshops increased participants’ disaster preparedness knowledge and were particularly effective in fostering social connections. The study underscored that disaster planning was more effective when strong community connections and social relationships were in place. These findings, along with those from other studies [30], emphasize the importance of a community network and education provided in immigrants’ native language, aligning with the results of the current study.

The control group in our study, which did not receive a disaster preparedness intervention but only completed the K-HEPI instrument, also showed elevated posttest K-HEPI scores. This finding suggests that the instrument itself may function as a form of intervention by exposing respondents to new knowledge about disaster preparedness, potentially motivating them to begin disaster planning.

We were not able to randomly assign participants to groups, and all participants from the same site were in the same group, limiting external validity. We applied strict inclusion and exclusion criteria to maximize group homogeneity and control confounders, reducing threats to internal validity. However, the study was at risk for selection bias, as participants had to contact the study team to enroll. Social desirability bias was minimized by having participants self-report their household disaster preparedness using an anonymous survey rather than verbally to study team members.

To further address the internal validity, temporality was established by confirming participants were unprepared for disasters before the intervention, allowing for a measurable change in preparedness post-intervention. Differences in participant characteristics between the experimental and control groups were disclosed, and attrition rates were low in both groups. Information bias was mitigated through random audits of 40% (*n* = 80) of the experimental group and 20% (*n* = 40) of the control group data transcription. Additionally, the statistician was blinded to group assignment during analysis.

History bias was a possible concern, as the study setting regularly experiences extreme weather events. During initial data collection, participants were asked whether they had experienced a disaster, allowing this variable to be controlled as a moderator or covariate in the analysis. To address history bias, we tracked local disasters, extreme weather events, and public health emergencies during data collection. Events recorded in NYC during this period included five flood watches, two flash flood warnings, one winter storm warning, one winter weather and travel advisory, a 4.8 magnitude earthquake with several minor aftershocks, and three heat advisories. During all these events, NYC Emergency Management officials disseminated risk messages and preparedness actions through email, text messages, and social media. Both experimental and control-group participants may have been exposed to these mass media messages during data collection.

Instrumentation bias was mitigated by field testing the K-HEPI prior to its use in this before-after study. However, testing bias remains a legitimate threat to the internal validity of the findings for both the experimental and control groups. While there is some evidence that exposure to the HEPI increases participants’ knowledge and awareness of household emergency preparedness [24], there is no current evidence to suggest that this increased knowledge translates into action.

The K-HEPI and N-TORM intervention would benefit from being tested over longer periods. To support this goal, participants in this study were asked for consent to be recontacted after the study’s completion. If an extreme weather event or disaster occurs within 5 years, we will follow up with consenting participants to examine the relationship between disaster preparedness and outcomes such as surviving without rescue or external assistance and survival without acute exacerbations of chronic illness or changes in baseline functional status.

Further research is needed to develop disaster preparedness interventions tailored to high-risk subgroups within Korean immigrants, such as cancer survivors, individuals with chronic diseases, and those with disabilities. These populations face unique challenges during emergencies, including medical needs and limited mobility. Understanding their specific barriers and needs will inform the creation of specialized preparedness strategies.

Additionally, it is also advisable to investigate disaster preparedness education for community workers and healthcare providers serving immigrant populations. These professionals play a role in supporting at-risk groups but may lack the training necessary to address the challenges faced by immigrants. Enhancing their preparedness may strengthen the resilience of immigrant communities.

While numerous disaster preparedness materials exist, few specifically address the unique vulnerability of immigrant populations, who often face language barriers, cultural differences, and limited access to resources [27]. This study demonstrates that the English HEPI and N-TORM intervention can be successfully translated into another language while maintaining cultural relevance, highlighting its potential for adaptation among diverse immigrant communities. Tailored disaster preparedness interventions delivered in their native language are critical to address these gaps. Research also highlights the importance of leveraging social networks within communities to enhance preparedness efforts. Future research should explore the adaptation and implementation of the HEPI and study protocol in other immigrant populations to assess its broader applicability and effectiveness. Researchers interested in replication are encouraged to contact the corresponding author for access to the HEPI and study materials. Community centers or local organizations, where immigrants often have strong ties, are ideal locations for delivering these interventions. Utilizing trusted community resources can enhance program effectiveness and improve the safety of immigrant populations during emergencies.

## Electronic supplementary material

Below is the link to the electronic supplementary material.

Supplementary file1 (DOCX 16 KB)

## Data Availability

The data for this study has not been approved to be shared beyond the study team.

## Appendix

### Literature Search Strategies

The first search was delimited to peer-reviewed academic journal articles published between September 2001 and August 2022, with an abstract available, and in the English language. Simultaneous searches of the Academic Search Complete, MEDLINE Complete, CINAHL Complete, PubMed, PsycINFO, Social Sciences Full Text, Health and Psychosocial Instruments, and Google Scholar databases were conducted in July and August of 2022. Eligibility criteria included literature discussing the disaster experience of Asian immigrants in New York City (NYC) compared to non-Asian immigrants in NYC with property destruction, morbidity, mortality, and need for rescue and/or disaster relief assistance. The following keyword combinations were used: “pandemic OR epidemic OR outbreak OR covid-19 OR coronavirus OR hurricanes OR storm OR heat AND Asian AND NYC or New York City or New York,” “hurricanes AND Asian AND NYC or New York City or New York.” After removing duplicates, seven articles met eligibility criteria from the 117 initial results. Another eight articles were identified through Google Scholar, resulting in 15 articles that met inclusion criteria.

In August 2022, a second review of the literature was conducted to determine how Asian immigrants in NYC have been educated to improve knowledge or skills of evacuation, communication plans, and household disaster supply kits to prepare for disasters and extreme weather events. The search was delimited to full-text peer-reviewed academic journal articles published between January 2001 and August 2022 and with an abstract available. CINAHL Subject Headings were used to find appropriate terms and synonyms of keywords. Additionally, Medline MeSH Headings were consulted to retrieve the latest terminology to search the database. Concurrent searches of APA PsycArticles, APA PsycInfo, CINAHL Complete, MEDLINE Complete, and Social Sciences Full Text databases were conducted. To narrow down the target population, the following search was applied: “Asian OR Cambodian OR Chinese OR Filipino OR Hmong OR Japanese OR Korean OR Laotian OR Thai OR Vietnamese AND immigrant OR emigrant OR migrant OR non-native” AND “New York City OR NYC OR Brooklyn OR Queens OR Manhattan OR Bronx OR Staten Island” AND “individual AND disaster OR emergency OR extreme weather AND preparedness OR planning,” “household OR citizen AND emergency OR disaster AND preparedness,” “disaster OR emergency AND supplies OR kit OR drill OR education,” “disaster OR emergency OR extreme weather.” The search was then limited to keywords in the abstracts, which reduced results from 4837 to 657. After removing duplicates, 265 articles’ titles, subjects, and/or abstracts were screened, and no articles were found to be relevant to the disaster preparedness of Asian immigrants in NYC.
