# Supplementary material for: A Home Disaster Preparedness Intervention Study with Korean American Residents in New York City
Source: J Urban Health. 2025 Mar 31;102(2):332–43. doi: 10.1007/s11524-025-00974-z (PMC12031703; doi:10.1007/s11524-025-00974-z)
Supplement: Supplementary file 1 — Supplementary file1 (DOCX 16 KB) [file 11524_2025_974_MOESM1_ESM.docx]

**Online Figures and Tables**

| **Table 3**  *Participant Comments About the Intervention* |
| --- |
| Until now, I wasn’t sure what to do and felt insecure. |
| [N-TORM intervention] was good. |
| I’ll remember the important instructions. Thank you. |
| It was nice to prepare radio, emergency contacts, and emergency food supply. It was also a good opportunity to think about various situations. |
| The class was very beneficial to me. |
| I also recommend preparing a disaster preparedness kit inside moving bags. |
| Before preparing the kit, how about a [KCS] worker purchase the items in bulk and distribute them? The condition is that each person should take responsibility for the cost. |
| 1) I hope there is a way that all family members can attend the class so we can prepare together. 2) I hope we can listen to this through YouTube or text messages. 3) I hope we can see a safety video placed in areas where people are gathered, like in buses, trains, stations. |
| I wish the class can also include a disaster movie. |
| I was able to organize bank related financial documents. |
| Thank you for teaching me about the disaster preparedness plan, which I only thought about. I learned a lot while searching for items related to the disaster preparedness kit. I learned about personal tents and various equipment. Thank you so much. |
| It would be more helpful if you can also provide a waterproof folder. |
| It is a good tip. |
| I wish you could give more specific explanations and examples about emergency food supplies. |
